# Recovery of mycorrhizal fungi from wild collected protocorms of Madagascan endemic orchid *Aerangis ellisii* (B.S. Williams) Schltr. and their use in seed germination in vitro

**DOI:** 10.1007/s00572-020-00971-x

**Published:** 2020-06-14

**Authors:** Jonathan P. Kendon, Kazutomo Yokoya, Lawrence W. Zettler, Alison S. Jacob, Faye McDiarmid, Martin I. Bidartondo, Viswambharan Sarasan

**Affiliations:** 1 Royal Botanic Gardens, Kew, Richmond, Surrey, TW9 3DS UK; 2 Department of Biology, Illinois College, 1101West College Avenue, Jacksonville, IL 62650 USA; 3 Faculty of Natural Sciences, Department of Life Sciences, Silwood Park, Imperial College London, London, UK

**Keywords:** *Ceratobasidium*, Detritus, Symbiotic germination, Mycotrophy, Conservation

## Abstract

Orchid mycorrhizal fungi (OMF) are critical for seed germination and maintaining natural populations of orchids, yet the degree of specificity of most orchids to their mycorrhizal associates remains unknown. Many orchids are at risk of extinction, whether generalists or specialists, but orchid species of narrow fungal specificity are arguably under increased threat due to their requirement for specific fungal symbionts. This study characterises the fungi associated with *Aerangis ellisii*, a lithophytic orchid from a site in the Central Highlands of Madagascar. Culturable OMF isolated from spontaneous protocorms of this species from the wild were used for seed germination. In vitro germination and seedling development of *A. ellisii* were achieved with fungi derived from *A. ellisii* and an isolate from a different *Aerangis* species 30 km away. The significance of these findings and their importance to conservation strategies for this species and other *Aerangis* spp. is discussed. These results have important implications for the conservation of *A. ellisii* populations in Madagascar.

## Introduction

Madagascar is a well-known biodiversity hotspot to which 90% of its 1000 orchid species are endemic (Tyson 2000). What was once a continuum of natural vegetation blanketing the world’s 4th largest island has been converted mostly to farmland except in the most remote and inaccessible areas. Deforestation and habitat loss continue resulting in a patchwork of isolated orchid communities that remain separated from one another by land modified by humans and managed with fire (Harper et al. 2007). Considering that orchids may be especially vulnerable to acute environmental changes because of their extreme dependency on other co-habiting organisms (pollinators and mycorrhizal fungi), the conservation of these unique plants is both complicated and challenging relative to other angiosperms. Orchids on isolated rocky outcrops in the Central Highlands of Madagascar (CHM), for example, appear to suffer from inbreeding depression. Their specific pollinators (e.g. hawkmoths) may be less common and/or they must fly considerable distances to forage on nectar, possibly explaining why few capsules are produced among the species that cater to moths (e.g. *Aerangis*, *Angraecum*), and why a high proportion of their seeds often lack embryos (Kendon et al. 2017). Thus, with limited cross-pollination, fewer spontaneous seedlings are generated per year resulting in steady population decline over time.

**Fig. 1:**
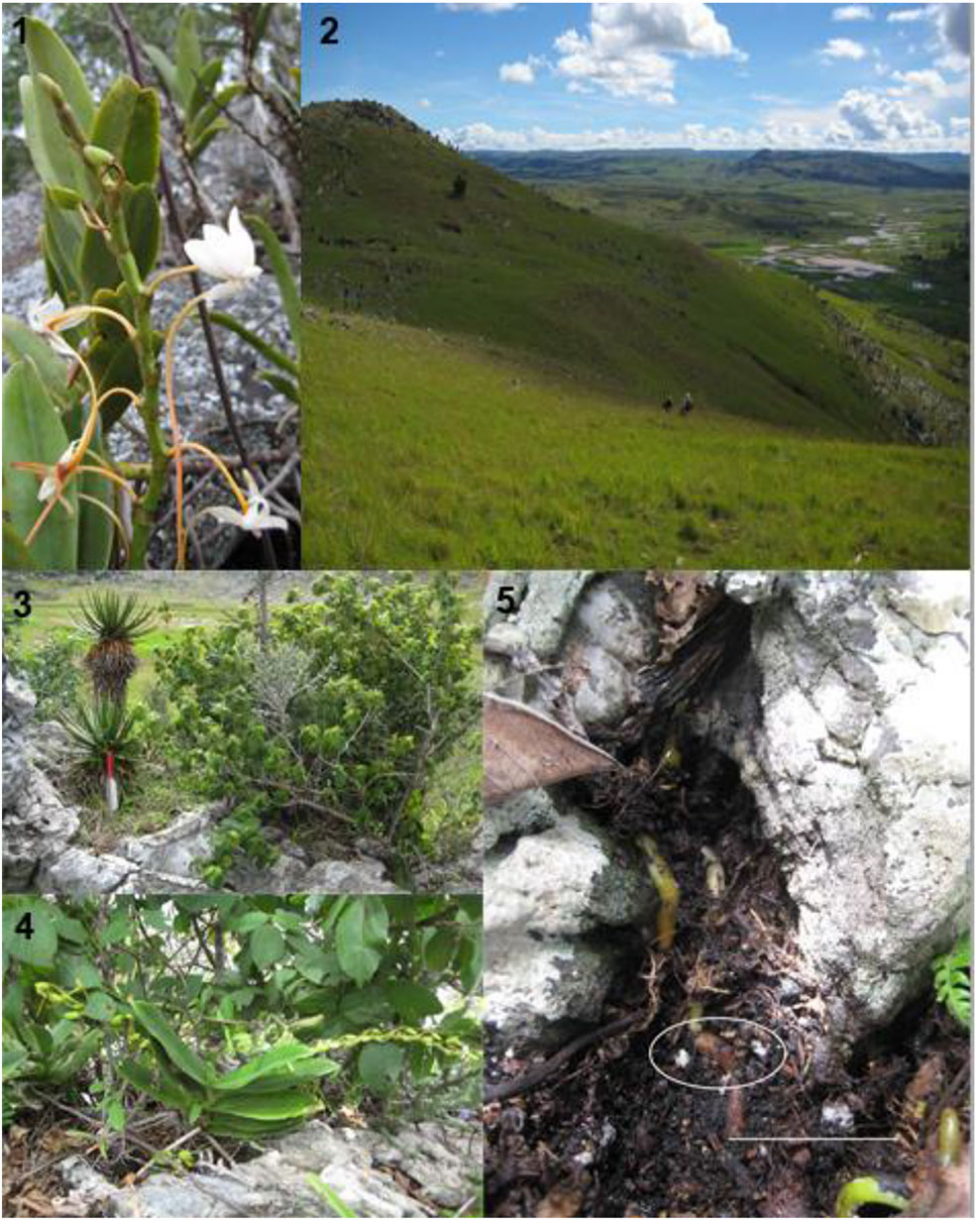
*Aerangis ellisii*, its habitat and protocorms found in situ. 1, inflorescence showing long nectar spur; 2, montane grassland with isolated rocky outcrops; 3, marble substrate and associated plant species at the site; 4, mature individual of *Aerangis ellisii* with two unopened inflorescences; 5, crevice containing roots of adult plant and white/greenish white protocorms (circled). Scale bar 5 cm

One such species in this predicament is *Aerangis ellisii* (B.S. Williams) Schltr. (Fig. 1) which grows as an epiphyte of humid evergreen forest as well as a lithophyte of granite rocky outcrops among xerophytic vegetation (Cribb and Hermans 2009). The species is pollinated by hawkmoths, although pollination events are infrequent and occur within a range of only 5 m (Nilsson et al. 1992). In the typical rocky inselberg habitat, *A. ellisii* appears to suffer from inbreeding depression possibly because much of the land lying between hilltops has become denuded of primary vegetation needed to support its pollinator(s). Nevertheless, *A. ellisii* is a long-lived species that occasionally produces capsules containing viable seeds. Where these seeds germinate in the rocky landscape remains a perplexing question that must be resolved if *A. ellisii* and other lithophytic orchids are to ultimately be conserved. Once protocorms and early seedlings are pinpointed, identifying the mycorrhizal fungi linked to seed germination and seedling development would then be possible. To our knowledge, no such studies have been reported for lithophytes in Madagascar or other parts of the world.

Orchids have been termed ‘specialists’ or ‘generalists’ depending on the degree by which they depend on select biotic agents needed to complete their life cycle (Otero et al. 2002; Swarts and Dixon 2009; Shefferson et al. 2005). All subfamilies of Orchidaceae typically form associations with basidiomycetes collectively referred to as rhizoctonias (e.g. *Tulasnella*, *Ceratobasidium*, *Thanatephorus* and *Serendipita = Sebacina*), and several studies (e.g. Zettler and Hofer 1998; McCormick et al. 2006; Girlanda et al. 2011) have revealed that some orchids display a high level of strain-to-species specificity towards these fungi, orchid mycorrhizal fungi (OMF). By targeting a narrow group of OMF, specialist orchids in a small area are more vulnerable to extinction should the fungi they depend on become scarce. Under this scenario, specialist orchids already handicapped by inbreeding depression would conceivably be at the highest risk of extinction.

During fieldwork conducted in January 2015 within the CHM, we found protocorms and seedlings of *A. ellisii* in their natural habitat. This discovery provided a unique opportunity to learn more about the specific germination requirements of this species linked to a rocky substrate and to ascertain whether *A. ellisii* is a fungal generalist or specialist. In this paper, we describe the germination site for this orchid, the protocorms and the peloton-forming fungi they harboured, and the results of in vitro symbiotic seed germination experiments using the fungi recovered to verify their link as mycorrhizal associates. Our goal is to enhance the conservation of *A. ellisii* and other rare lithophytic orchids by understanding seed germination requirements leading to more effective propagation from seed.

## Materials and methods

### Study site and field collection

Fieldwork in Madagascar took place during January 2015 in the Itremo Massif Protected Area of the CHM, with an exploratory trip taken to a site near the village of Mahavanona, Ambatofinandrahana district, Amoron’i Mania region (20° 35′ 11.1″ S 46° 49′ 25.9″ E). This mountainous area consists of grassland interspersed with rocky outcrops (Fig. 1). Burning of grassland is practiced at lower elevations, providing cattle with fresh growth, and some of these fires spread uphill to areas not used for grazing. The corresponding negative effect on orchids has been documented (Whitman et al. 2011), and evidence of recent widespread burning was observed on this trip. The most substantial-sized plants in the area were *Aloe cipolinicola* (H.Perrier) J.-B.Castillon & J.-P.Castillon, sparsely distributed, invasive *Pinus* spp. and solitary palms (Arecaceae). Quarrying activity for marble had resulted in damage to some of the orchid habitats, both directly by removing the rock substrate and indirectly through erosion of the ground nearby with heavy machinery. There was also evidence of illegal collecting of mature Angraecoid orchids that had been detached from exposed rock, leaving behind roots anchored in crevices. Although some epiphytic orchids were present at the site mostly affixed to the bark of sparse woody vegetation, most of the orchids consisted of lithophytes (e.g. *Jumellea*, *Aerangis*) and terrestrials (e.g. *Habenaria*, *Cynorkis*, *Eulophia*). All *A. ellisii* plants were rooted on exposed rock (marble) as lithophytes at a lower elevation within the valley (Fig. 1). No other *Aerangis* species were present, either in the vicinity of the collecting site or in the areas travelled on foot during the collecting day. Other flowering plants in the vicinity included *Aloe cipolinicola*, *Euphorbia stenoclada* Baill. and *Grewia* sp.

Special emphasis was made to locate and obtain spontaneous protocorms or seedlings as a means of recovering and identifying the mycorrhizal fungi responsible for seed germination, using previous fieldwork sites as a model (Yokoya et al. 2015; Rafter et al. 2016). All samples were collected under permit by the Department de l’Eau et Foret, Madagascar, under Millennium Seed Bank Partnership guidelines (Way 2003). Within 2 m of each mature plant, especially those with dehiscent capsules from the previous year, the surrounding substrate (rock surfaces and crevices) was carefully inspected for seedlings using a hand-lens where necessary. In a humus-filled rock crevice measuring 20–30 cm in width and 5–10 cm deep, a spatula was used to gently scrape away organic matter that revealed several small (< 1 cm) pale white protocorms (Fig. 1). We define the structures collected as protocorms following the type VIII isobilateral growth classification of many orchids in tribe Vandaea by Clements (1995). Out of four crevices, within 1-m radius of the mature plants, protocorms were discovered. However, protocorms were not found in any of the three adjacent crevices that we inspected. Live protocorms from a sample of the existing population were collected and transported to the UK (Kew) and USA (Illinois) using the methods described by Yokoya et al. (2015). This included a total of six protocorms that were placed in sterile glass vials along with a small quantity of soil. All protocorm samples were then transported back to Kew Madagascar Conservation Centre (KMCC), Antananarivo, in insulated coolers within 1 week of collection (Zettler et al. 2017). Prior to departure from Madagascar, soil associated with the protocorm was tested for various chemical parameters using a test kit (LaMotte, MD, USA). After chemical testing, soil was then discarded in Madagascar. Samples were packaged and transported as in Yokoya et al. (2015).

### Fungal documentation, isolation and identification

Upon arrival at the laboratory, the samples were promptly (< 24 h) processed for isolating fungi present in the tissues. Six protocorms were sectioned (labelled 1–6) starting at the base upward until there was no sign of fungal colonization. In sections where pelotons were observed, clumps of cells from each section were teased out using a sterile scalpel assisted by dissection microscope and plated directly onto Fungal Isolation Medium (FIM; Clements et al. 1986) supplemented with 100 mg/l streptomycin sulphate to isolate and identify the peloton-forming fungi.

Culturing of the pelotons was achieved in vitro according to Yokoya et al. (2015). Due to the high pH of the substrate (7.8) as tested in Madagascar, FIM media, one at the pH of 5.5 and the other adjusted to pH 7.5 were used. Hyphal tips originated from pelotons were transferred to fresh FIM of the same pH.

Using light microscopy, fungus mycelia were inspected during a 2–4-week period for morphological characteristics matching published descriptions of *Rhizoctonia*-like fungi (Currah et al. 1997). Cultures that were provisionally identified as potential mycorrhizal associates (e.g. Ceratobasidiaceae) were retained for further study. Pure cultures of fungi from protocorms and mature roots were positively identified using DNA sequencing as described in Yokoya et al. (2015). Briefly, DNA from fresh mycelia was extracted in 96-well plates using the Extract-N-Amp™ Plant Tissue PCR Kit (Sigma Aldrich, UK). PCR amplification of the ITS region using primer ITS1F with ITS4 and ITS1 with ITS4-tul (White et al. 1990; Gardes and Bruns 1993; Taylor and McCormick 2008) was followed by Sanger sequencing using the same forward and reverse primers. The forward and reverse sequences were checked for accuracy and consensus and compared with database sequences using BLAST (National Center for Biotechnology Information, Bethesda, MD, USA). Sequences that matched *Rhizoctonia*-like fungi were aligned and grouped into operational taxonomic units (OTUs) based on a conservative similarity threshold of 95%. Representative sequences of each OTU were used to requery the GenBank database using BLAST and deposited in GenBank. Orchid mycorrhizal fungal isolates were cryopreserved at RBG Kew under for future reference and use in conservation.

### Molecular confirmation of species identification of putative *A. ellisii* protocorms

The DNA of protocorms that yielded *Rhizoctonia*-like fungi was extracted and sequenced to confirm their putative species identification by matching the sequence of the chloroplast DNA region *trnL-F* to available sequences in GenBank database. DNA was extracted from protocorms using Sigma Extract-N-Amp™ Plant Tissue PCR Kit (Sigma Aldrich, St. Louis, MO, USA) or with a modified CTAB (cetyltrimethylammonium bromide) protocol (Doyl and Doyle 1987) followed by chloroform/isoamyl alcohol (24:1) extraction and precipitation in isopropanol. The *trnL-F* sequences were amplified using primer combinations c with d for the *trnL* intron and e with f for the *trnL-F* intergenic spacer (Taberlet et al. 1991) as described previously (Yokoya et al. 2015). The PCR products were cleaned using QIAquick® columns (Qiagen Inc., East Crawley, UK) and sequenced as described above for fungus ITS sequencing.

### Seed germination

**Fig. 2:**
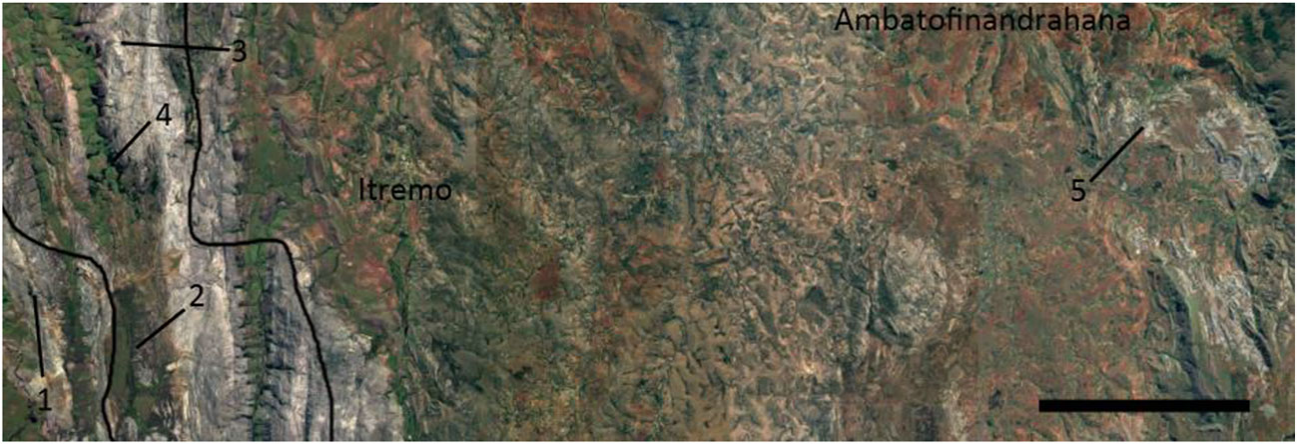
Collection sites of orchid material from which OMF were isolated, near the settlement of Itremo and the town of Ambatofinandrahana. Borders of the core protected area, Itremo NAP (Nouvelle Aire Protégée), are shown. Sites 1 to 4 are locations of orchid material producing OMF from 2013 field work. Site 5 is the location of the 2015 discovery of *Aerangis ellisii* protocorms from which OMF were isolated. Scale bar 5 km

Mature seeds of *A. ellisii* were used in symbiotic germination experiments using standard protocols (Clements et al. 1986). The seeds used were collected at site 2 (Fig. 2) from a mature, nearly dehiscent capsule from a natural population within the CHM during a previous trip (Kendon et al. 2017). Capsules were collected and stored in paper envelopes inside a Ziplock bag containing silica gel beads as a desiccant. At RBG Kew capsules were cut open and seed transferred to vials for further drying and cold storage according to Seaton and Pritchard (2003). Prior to cold storage at 4 °C, seeds were tested for viability by sowing on asymbiotic medium P6668 (Sigma Aldrich, UK) (Ramsay and Dixon 2003) using 0.5% sodium dichloroisocyanurate as a surface sterilant (Sarasan et al. 2006) and incubated at 22 °C ± 2 °C and 16/8 h photoperiod at 20 μmol m^−2^ s^−1^. Germination occurred 6 months after sowing, and this seed batch had a very low proportion of full seed, but many of the full embryos were viable.

For symbiotic germination, seeds were surface sterilised as above and sown onto oatmeal agar (OMA, Clements et al. 1986) minus minerals and sucrose in clear 5 cm Petri dishes. The medium had been autoclaved at 121 °C for 15 min. An additional variable tested was use of agar-free liquid oatmeal medium, to determine whether germination could be improved by full medium contact with the seed.

Seeds were inoculated with different fungal isolates that had been stored on OMA at 4 °C in 2013 work (Yokoya et al. 2015) and one isolate from an *Aerangis ellisii* protocorm collected in 2015 (Table 1). A 1 cm^3^ cube of agar containing the mycelium from each fungal isolate was added to each Petri dish (one OMFisolate per dish), and each Petri dish was sealed with ‘Parafilm M’ (Pechiney Plastic Packaging, Menasha, WI, USA). Control replicates of seeds were sown on OMF-free OMA medium. Cultures were incubated at 22 °C ± 2 °C and 16/8 h photoperiod under cool white fluorescent light at 20 μmol m^−2^ s^−1^ and inspected every 4 weeks for signs of germination (= rupture of the testa and emergence of protocorm). Seeds in the act of germinating were then scored every 4 weeks for 6 months using the method of Rafter et al. (2016) followed by emergence of first leaf as part of seedling development. Four replicate plates were set up for each treatment with a minimum of 20 embryo-bearing seeds per replicate (the seed batch had a very low percentage of full seeds).

**Table 1:** Inoculations made in vitro for seeds of *Aerangis ellisii*. Fungal isolates had been stored in oatmeal agar (OMA) at 4 °C until use. OTUcer2^2^ was isolated in 2015; all others in 2013 (Yokoya et al. 2015). For field site information, see map (Fig. 2)

Orchid mycorrhizal fungus ID	Field site	Medium/fungus
OTUcer2^2^	5	OMA + isolate from *Aerangis ellisii* protocorm
OTUcer1	4	OMA + *Ceratobasidium* isolated from *Aerangis* sp. gallery forest epiphyte seedling
OTUcer2^1^	2	OMA + *Ceratobasidium* isolated from *Aerangis ellisii* seedling
OTUcer3	1	OMA + *Ceratobasidium* isolated from *Aerangis* sp*.* gallery forest epiphyte seedling
OTUtul2	2	OMA + *Tulasnella* isolated from *Benthamia cinnabarina* mature plant
OTUtul3	4	OMA + *Tulasnella* isolated from *Cynorkis purpurea* seedling
OTUtul5	2	OMA + *Tulasnella* isolated from *Tylostigma* sp.
OTUseb1	4	OMA + *Serendipita* (= *Sebacina*) isolated from *Cynorkis purpurea*
P6668	–	Phytamax orchid medium (Sigma Aldrich, UK)
Control	–	Oatmeal agar (OMA)

### Statistical analysis

Statistical analysis was performed in R 3.4.3. Using the survival package, a Kaplan-Meier estimator of survival function was used to assess (a) differences in rate of protocorm production between liquid versus agar media and (b) rate of protocorm development to stage 4 between the three isolates (McNair et al. 2012). Analysis of variance (ANOVA) followed by Tukey HSD was performed in R using the Agricolae package to assess differences in production of stage 4 protocorms and seedlings between the isolates.

## Results

### Protocorm description

A total of 20 protocorms were uncovered in a single rock crevice (pocket) at a depth of between 1 and 10 cm and in proximity (1–2 cm) to roots of a mature *A. ellisii* plant growing on a bare rock surface (Fig. 1). This plant was one of at least 10 mature *A. ellisii* individuals clustered into a small area (10 × 10 m) with the protocorms in direct contact with moist organic debris. The protocorms were milky or greenish white in colour with slightly yellowish tint at the base displayed clear polarity, i.e. a well-defined base and tip, despite not all having shoot or root initials. Protocorms were provisionally identified as belonging to *A. ellisii* based on morphology, i.e. the shape, dimension, texture, and colour closely matched protocorms of this species cultivated asymbiotically in vitro at RBG Kew. This initial identification was confirmed by the molecular techniques.

Two chloroplast DNA regions were amplified and sequenced from each of the six protocorms. All protocorms had identical *trnL* intron and *trnL-F* intergenic spacer sequences and matched *A. ellisii* (KF558204) 100% in GenBank.

**Fig. 3:**
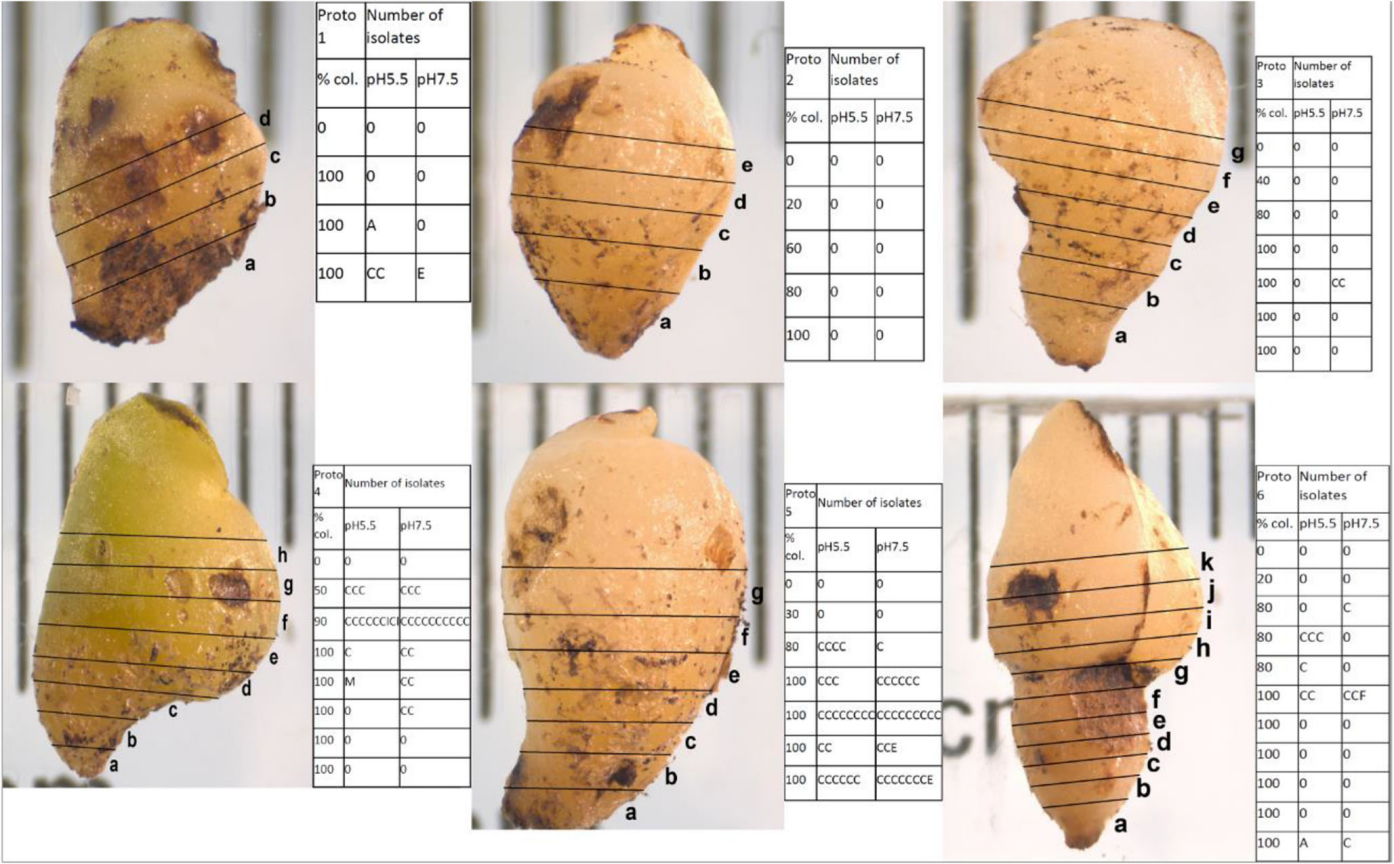
Section sites of six protocorms of *Aerangis ellisii* showing approximate percentage cell colonization by pelotons (% col.) observed under light microscope and numbers of individual culturable endophytic fungi isolated in either pH 5.5 or pH 7.5 FIM. *C*, *Ceratobasidium* OTUcer2^2^; *A*, *Arthroderma*; *E*, *Exophiala oligosperma*; *Cl*, *Cladosporium*; *M*, *Myrothecium inundatum*; *F*, *Fusarium*. Scale bar = 1 mm. Protocorm numbering (‘Proto’) denotes likely order of growth stages, i.e. 1 least developed, 6 most developed. Protocorm 6 is the only one to have initiated a vascular root

Thin sections of the protocorms revealed that all were heavily colonized by pelotons, especially the basal half of the protocorm (Fig. 3). At the base of all six protocorms, 100% of cells were colonized by OMF tapering off to < 50% colonization towards the middle region. The apical region lacked any traces of fungi. Thus, a highly defined boundary was visibly evident between colonized and uncolonized tissue. Externally, the lower mycotrophic region showed a slightly orange coloration. In some sections, morphological differences were noted between pelotons at the base compared with those towards the centre (upper extent of peloton layer), i.e. those at the base appeared opaquer and more rounded, whereas those at the top of the colonized zone were more transparent and diffuse.

More than half of the pelotons teased out of protocorm sections yielded fungus colonies. Of the pelotons that failed to grow, most were from the upper and lower zones of colonization. In culture, these fungi displayed morphological characteristics (e.g. hyphal growth rates, colony colour, barrel-shaped monilioid cells) that closely matched published descriptions for *Ceratobasidium* (Basidiomycota, Cantharellales, Ceratobasidiaceae; anamorphs formerly *Ceratorhiza*), a ubiquitous mycorrhizal associate in the *Rhizoctonia* complex (Currah et al. 1997; Zettler and Corey 2018).

### Molecular identification of peloton-forming fungi

DNA sequencing of the ITS regions of the peloton-forming fungi isolated from three of the six protocorms confirmed their identity as belonging to the family Ceratobasidiaceae. The protocorm-derived isolates of OMF were of the same OTU as the Ceratobasidiaceae OTUcer2 isolated from *A. ellisii* seedling roots as described in Yokoya et al. (2015). These protocorm-derived isolates are therefore referred to herein as OTUcer2^2^ and the seedling-derived isolates as OTUcer2^1^. No other OMF taxa, namely *Sebacina* and *Tulasnella*, were found among the cultures isolated from the protocorms.

Other types of non-peloton-forming fungi were isolated from protocorm sections. These consisted of common saprophytes identified by DNA sequencing as *Arthroderma* sp., *Cladosporium* sp., *Exophiala oligosperma*, *Fusarium* sp. and *Myrothecium inundatum*. While agar pH (5.5 versus 7.5) had little effect on the growth rates of *Ceratobasidium*, pH did appear to influence recovery of the saprophytes (Fig. 3).

### In vitro symbiotic seed germination

**Fig. 4:**
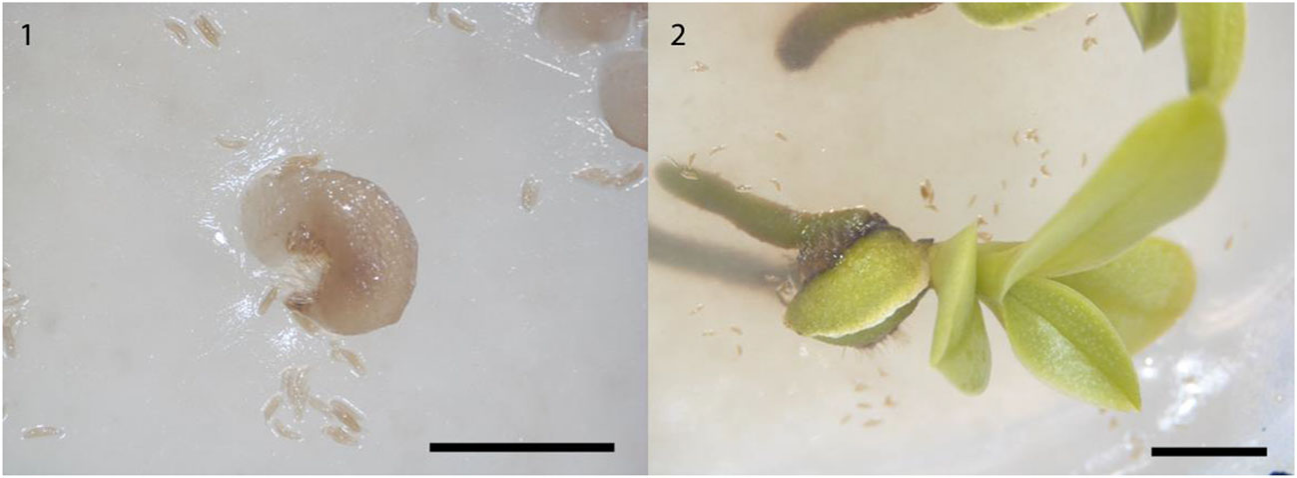
Symbiotically raised protocorm (1) and seedling (2) of *Aerangis ellisii*. Scale bar = 5 mm

Seeds of *Aerangis ellisii* inoculated with OMF *Ceratobasidium* acquired from three sources (OTUcer3 from *Aerangis* sp. root, OTUcer2^1^ from *A. ellisii* seedling root, OTUcer2^2^ from *A. ellisii* protocorm) germinated in vitro after 8 weeks in incubation, and seedlings with leaves and vascular roots were produced thereafter (Fig. 4).

Germination and seedling development were observed in only two of the OTUs tested. The germination and seedling development were higher than that of the control asymbiotic medium (Table 2).

**Table 2:** Fungal isolates tested for symbiotic germination of *Aerangis ellisii*, including conversion rate from germinated protocorm to seedling in vitro. OTUcer2^1^ is seedling-derived, and OTUcer2^2^ is protocorm-derived, both from *A. ellisii*. P6668 is a control asymbiotic medium (Sigma Aldrich, UK). OMA is oatmeal agar control with no mycorrhizal fungus inoculum

Orchid mycorrhizal fungus ID	Germination by 6 months (% ± SE)	Seedlings by 6 months (% ± SE)	Conversion rate (% ± SE)
OTUcer3	60.0 ± 10	47.5 ± 16	79.2 ± 13
OTUcer2^1^	57.4 ± 17	18.9 ± 12	32.9 ± 13
OTUcer2^2^	39.9 ± 15	5.0 ± 4	12.5 ± 8
OTUcer1	0	0	0
OTUtul2	0	0	0
OTUtul3	0	0	0
OTUtul5	0	0	0
OTUseb1	0	0	0
Control 1 (P6668, no fungus)	2.3 ± 1	1.3 ± 1	47.8 ± 2
Control 2 (OMA, no fungus)	0	0	0

**Fig. 5:**
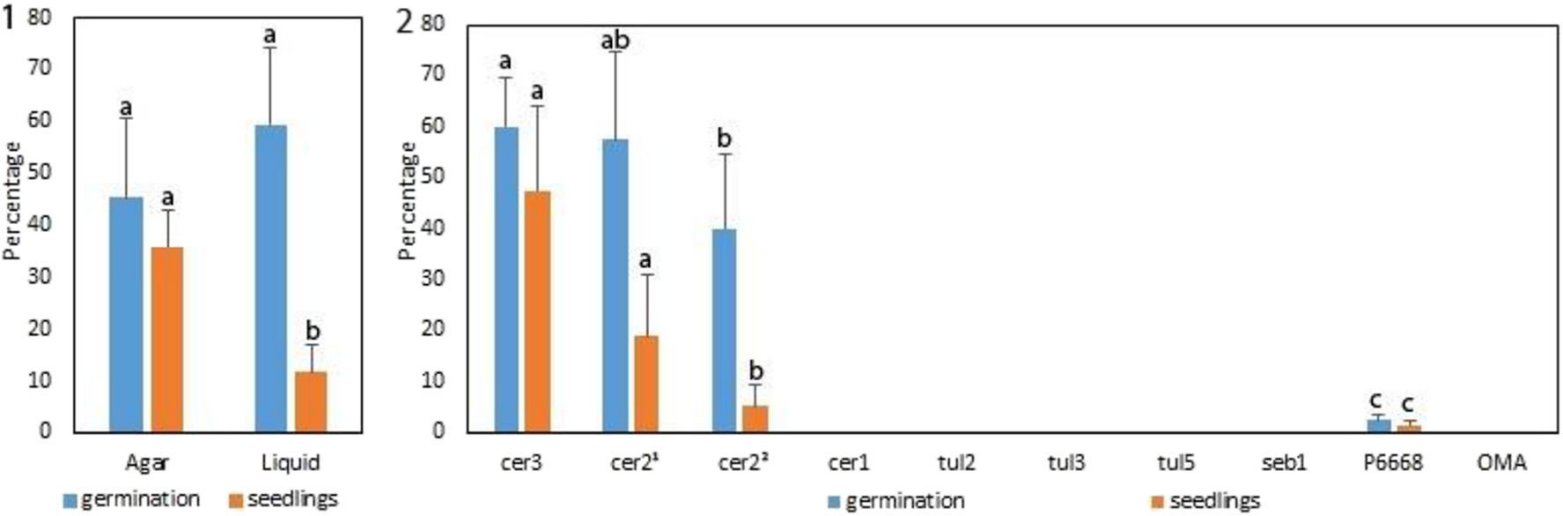
Symbiotic germination and seedling development in *Aerangis ellisii* cultured under different conditions 6 months after sowing and inoculation. 1, agar vs. liquid medium combining results for all isolates tested; 2, three *Ceratobasidium* isolates, OTUcer3 from *Aerangis* sp.; OTUcer2^1^ from *A. ellisii* seedling, OTUcer2^2^ from *A. ellisii* protocorm in current study and a selection of other OMF OTUs from CHM. Germination and seedling values with the same letter are not significantly different (*P* > 0.05) by Tukey’s HSD. Germination and seedlings percentage for P6668 (asymbiotic control) were not significantly different from those of uninoculated OMA, cer1, tul2, tul3, tul5 or seb1 (zero)

OTUcer3 germinated more seeds than OTUcer2^2^. Furthermore, more of the protocorms developed to seedling stage when inoculated with fungus OTUcer3 and OTUcer2^1^ compared with OTUcer2^2^ (Fig. 5).

No significant differences were noted between agar versus liquid medium on germination; however, protocorm progression to the seedling stage was markedly greater on agar (Fig. 5). There were no significant differences between liquid and agar media on seedlings that transitioned from stage 2 to stage 3 using the Kaplan-Meier estimator of survival function. However, more protocorms progressed to stage 4 on agar compared with liquid media as revealed by Kaplan-Meier and chi-squared test. ANOVA and Tukey’s HSD showed both OTUcer3 and OTUcer2^1^ were significantly more effective than OTUcer2^2^ in facilitating protocorm development to stage 4 seedlings. The uninoculated control produced zero germination or seedlings.

### Characterisation of mycorrhizal fungi from protocorms and seedlings

Three OTUs (OTUcer1–3) in this study were placed within the family Ceratobasidiaceae (Table 3). The sequences of OTUcer2^1^ and OTUcer2^2^ differed at five nucleotide loci over 665 nucleotides, a match of 99.2%. All five differences were substitutions to the alternative purine or pyrimidine.

**Table 3:** Operational taxonomic units (OTUs) of fungi isolated from *Aerangis ellisii* and their possible identities based on ITS sequences

OTU	Source species	Closest matches in GenBank	Identity (%)
OTUcer2^2^	*A. ellisii* protocorm	JF691430 uncultured Ceratobasidiaceae, *Jumellea rossii* root associated fungus, Reunion	94.55
OTUcer1	*A.* sp. 1 seedling	MK555327 Fungal sp. *Dendrobium exile* (Orchidaceae) root associated fungus, China?	95.09
OTUcer2^1^	*A. ellisii* seedling	JF691430 uncultured Ceratobasidiaceae, *Jumellea rossii* root associated fungus, Reunion	94.87
OTUcer3	*A.* sp. 2 seedling	MF992150 Ceratobasidiaceae sp., *Comparettia falcata* (Orchidaceae), C. America?	94.68

The closest matches in GenBank of all three Ceratobasidiaceae OTUs were also orchid-associated fungi. With reference to the two OTUs identified as successfully germinating seeds of *A. ellisii*, OTUcer2 is related to *Ceratobasidium* sequences from the orchid *Jumellea rossii*, epiphytic orchid endemic to Réunion (Martos et al. 2012).

## Discussion

To investigate which fungal isolates are capable of germinating *A. ellisii* seeds, we sampled 30 seedling/mature plants of *A. ellisii* and related species across three sites in Madagascar including other orchid species in their vicinity. Our results show that all culturable peloton-forming fungi from all species of *Aerangis* (*A. ellisii*, *Aerangis.* spp. and *A. punctata*) belong to Ceratobasidiaceae. Of these isolates, we have demonstrated that two Ceratobasidiaceae OTUs (OTUcer2^1^/cer2^2^ and OTUcer3) are successful in the symbiotic germination of *A. ellisii* seeds. It is noteworthy that the OTU isolated from *Aerangis.* sp. (OTUcer3) was capable of germinating and supporting the development of protocorms to seedling stage of *A. ellisii* seeds to a greater extent than isolates cultured from *A. ellisii* (OTUcer2^1^ and OTUcer2^2^)*.*

As far as we know, this is the first instance of naturally occurring protocorms of a lithophytic orchid being discovered. From our experiments, we conclude that *A. ellisii* has pronounced specificity towards its mycorrhizal associate. Only three of the 8 isolates tested produced germination and seedlings, while the remaining isolates plus uninoculated control failed to facilitate germination. This contrasts with other work that has found orchid seed germination proceeds in a generalist fashion and even without inoculation, while any specificity is noticed at progression from protocorm to seedling stage (Rafter et al. 2016; Meng et al. 2019). Such orchids may be colonized through the rhizoids. *Aerangis ellisii*, it is concluded, is not capable of early stage germination without its mycorrhiza and must be colonized at an earlier stage, perhaps through the suspensor as reported previously for other orchids (Rasmussen and Rasmussen 2009).

All isolates from *A. ellisii* protocorms found in the same rock crevice were of the same OTU as an isolate from an *A. ellisii* seedling acquired 30 km away. Strikingly, the ITS sequence of these two *A. ellisii* fungi had a 99.2% match, whereas the pairwise identity between the other Ceratobasidiaceae OTUs ranged from 86.2 to 87.4% (data not shown). This OTU was not found in any other orchid we collected in CHM among over 250 collections from sites in an area of over 2500 km^2^, highlighting the specificity of the relationship between *A. ellisii* and OTUcer2. The lithophyte’s host substrates were also different (quartzite versus marble [Moat and Smith 2007]) as were the pH values (7.8 versus 5.8) and associated plant species. Between these sites, the landscape appeared unsuitable for *A. ellisii* because it consisted of undulating grassland maintained for cattle and/or crops and was largely devoid of exposed rock.

The lithophytes are among the least understood of all orchids because few of the 27,000+ orchid species worldwide have adapted solely to life on bare rock surfaces. In orchid-rich Madagascar, however, lithophytes are abundant especially on the Itremo Massif of the CHM—home to many well-known *Angraecum* and *Aerangis* species with white flowers adapted to hawkmoth pollinators. In the case of *A. ellisii*, which is both epiphytic as well as lithophytic, preliminary assessments suggest an IUCN rating of Least Concern (LC) due to its wide distribution across the country (Landy Rajaovelona, pers. comm.), but it is included in Appendix I of CITES in recognition of its vulnerability through desirability to the trade UNEP-WCMC (Comps.) (2018).

Seeds of lithophytic orchids presumably germinate in rock crevices where mycorrhizal fungi would have access to plentiful moisture and organic matter. These ‘pockets’ of humus would then serve as a microsite for decomposition, and it is conceivable that orchid seeds would germinate and develop quickly into protocorms at a time when moisture levels are high, i.e. during the rainy season. This is supported by our findings that only 8 weeks after sowing; the seeds produced protocorms in vitro. Roots of mature *A. ellisii* were also present in rock crevices in close contact with moist organic matter. The protocorms of *A. ellisii* were uncovered near these roots, which may serve as a source of inoculum for seed germination as found elsewhere (Rammitsu et al. 2019). By germinating seeds of *A. ellisii* in vitro using this fungus, we can confidently conclude that this *Ceratobasidium* strain (OTU) is a specific mycorrhizal associate of *A. ellisii* in the natural rocky landscape.

Whether or not *A. ellisii* is a specialist orchid that relies on just one *Ceratobasidium* strain (OTU) beyond the CHM remains to be determined, testing with other primers and DNA regions would indeed be useful for future work as a means to detect additional fungi, but we are confident that *A. ellisii* is a specialist orchid for at least one strain of *Ceratobasidium*. This might be resolved by future work using samples from other habitats in provinces where populations of this orchid are distributed according to the latest survey (Landy Rajaovelona, pers. com.). *Aerangis ellisii* also exists as an epiphyte in humid evergreen forests in the northern and southern regions of Madagascar (Cribb and Hermans 2009); therefore, isolating and comparing fungi from its epiphytic niche would be an especially interesting study. As this study has shown, *A. ellisii* can utilize *Ceratobasidium* of a different OTU under laboratory conditions, i.e. from an epiphytic *Aerangis* species 30 km from the protocorm site. It is conceivable, therefore, that *A. ellisii* relies on a different group of *Ceratobasidium* strains as an epiphyte compared with a lithophyte.

Among the *Rhizoctonia*-like fungi that form mycorrhizal associations with orchids, members of the genus *Ceratobasidium* are known to produce polyphenoloxidases—enzymes aimed at lignin breakdown (Rasmussen 1995). Over time, the physical nature of uneven rocky surfaces would funnel organic (woody) debris and moisture from runoff into crevices forming deep pockets conducive to colonization and persistence of *Ceratobasidium*. Thus, it might be likely that *Ceratobasidium* serves as an important mycorrhizal associate of *A. ellisii* in Madagascar because of its ability to digest lignin trapped in rock crevices (Zelmer et al. 1996).

### Pollination, seed viability and germination

Under natural conditions, orchid pollination is often sporadic and may result in low fruit set (Nilsson and Rabakonandrianina 1988, Nilsson et al. 1992). Indeed, not only did we observe low fruit set for *A. ellisii* in Madagascar, a high proportion of seeds from the capsules collected lacked embryos that we attribute to infrequent pollination and/or inbreeding depression. Consequently, germinating this species from seed for conservation is problematic. In a previous study, a mere 10% of mature *A. ellisii* seeds sown on asymbiotic media germinated, but for immature seeds the percentage increased to ca. 70% (Kendon et al. 2017). In this study, symbiotic germination produced a greater number of seedlings than asymbiotic culturing. This outcome has a profound impact on conservation strategies because a small amount of wild-collected genetically diverse seeds can yield *A. ellisii* seedlings using a compatible mycorrhizal fungus (*Ceratobasidium*). We recommend using OMF derived from the same habitat, and ideally from the same species, to avoid reintroduction of unfit propagules (seedlings) and alien fungi.

### Further considerations

Global biodiversity hotspots like Madagascar hosts thousands of endemic orchids mostly in fragmented habitats with declining seed set and poor natural recruitment of seedlings. The IUCN Red List contains 37 species of lithophytes of which 86% are threatened by biological resource use around the world (Wraith and Pickering 2018). Therefore, this group of plants is high priority for conservation. As part of an integrated ex situ species recovery programme identification of putative mycorrhizal fungi for seed germination and development of symbiotic seedlings are critical as reported before (Zettler et al. 2001; Johnson et al. 2007; Zettler et al. 2007, Swarts and Dixon 2009; Rafter et al. 2016; Fay et al. 2018). This study highlights how a geographically isolated lithophytic orchid (*A. ellisii*) from a biodiversity hotspot can be successfully propagated symbiotically. The outcomes of this research have implications for the conservations of more than 20 other *Aerangis* spp. found in Madagascar and several other lithophytes from around the world.

## References

[CR1] Clements MA (1995) Reproductive biology in relation to phylogeny of the Orchidaceae especially the tribe Diurideae. PhD thesis, Australian National University

[CR2] Clements MA, Muir H, Cribb PJ (1986). A preliminary report on the symbiotic germination of European terrestrial orchids. Kew Bull.

[CR3] Cribb P, Hermans J (2009). Field guide to the orchids of Madagascar.

[CR4] Currah RS, Zelmer CD, Hambleton S, Richardson KA (1997). Fungi from orchid mycorrhizas. Orchid biology.

[CR5] Doyl JJ, Doyle JL (1987) A rapid DNA isolation procedure for small quantities of fresh leaf tissue. Phytochem Bull 19:11–15

[CR6] Fay MF, Feustel M, Newlands C, Gebauer G (2018). Inferring the mycorrhizal status of introduced plants of *Cypripedium calceolus* (Orchidaceae) in northern England using stable isotope analysis. Bot J Linn Soc.

[CR7] Gardes M, Bruns TD (1993). ITS primers with enhanced specificity for basidiomycetes – application to the identification of mycorrhizae and rusts. Mol Ecol.

[CR8] Girlanda M, Segreto R, Cafasso D, Liebel HT, Rodda M, Ercole E, Cozzolino S, Gebauer G, Perotto S (2011). Photosynthetic Mediterranean meadow orchids feature partial mycoheterotrophy and specific mycorrhizal associations. Am J Bot.

[CR9] Harper GJ, Steininger MK, Tucker CJ, Juhn D, Hawkins F (2007). Fifty years of deforestation and forest fragmentation in Madagascar. Environ Conserv.

[CR10] Johnson TR, Stewart SL, Dutra D, Kane ME, Richardson L (2007). Asymbiotic and symbiotic seed germination of *Eulophia alta* (Orchidaceae)—preliminary evidence for the symbiotic culture advantage. Plant Cell Tiss Org.

[CR11] Kendon JP, Rajaovelona L, Sandford H, Fang R, Bell J, Sarasan V (2017). Collecting near mature and immature orchid seeds for ex situ conservation: ‘in vitro collecting’ as a case study. Bot Stud.

[CR12] Martos F, Munoz F, Pailler T, Kottke I, Gonneau C, Selosse MA (2012). The role of epiphytism in architecture and evolutionary constraint within mycorrhizal networks of tropical orchids. Mol Ecol.

[CR13] McCormick MK, Whigham DF, Sloan D, O'Malley K, Hodkinson B (2006). Orchid–fungus fidelity: a marriage meant to last?. Ecology.

[CR14] McNair JN, Sunkara A, Frobish D (2012). How to analyse seed germination data using statistical time-to-event analysis: non-parametric and semi-parametric methods. Seed Sci Res.

[CR15] Meng YY, Shao SC, Liu SJ, Gao JY (2019). Do the fungi associated with roots of adult plants support seed germination? A case study on Dendrobium exile (Orchidaceae). Global Ecology and Conservation.

[CR16] Moat J, Smith P (2007). Atlas of the vegetation of Madagascar.

[CR17] Nilsson LA, Rabakonandrianina E (1988). Hawk-moth scale analysis and pollination specialization in the epilithic Malagasy endemic *Aerangis ellisii* (Reichenb. fil.) Schltr.(Orchidaceae). Bot J Linn Soc.

[CR18] Nilsson LA, Rabakonandrianina E, Pettersson B (1992). Exact tracking of pollen transfer and mating in plants. Nature.

[CR19] Otero JT, Ackerman JD, Bayman P (2002). Diversity and host specificity of endophytic Rhizoctonia-like fungi from tropical orchids. Am J Bot.

[CR20] Rafter M, Yokoya K, Schofield EJ, Zettler LW, Sarasan V (2016). Non-specific symbiotic germination of *Cynorkis purpurea* (Thouars) Kraezl., a habitat-specific terrestrial orchid from the Central Highlands of Madagascar. Mycorrhiza.

[CR21] Rammitsu K, Yagame T, Yamashita Y, Yukawa T, Isshiki S, Ogura-Tsujita Y (2019). A leafless epiphytic orchid, *Taeniophyllum glandulosum* Blume (Orchidaceae), is specifically associated with the Ceratobasidiaceae family of basidiomycetous fungi. Mycorrhiza..

[CR22] Ramsay MM, Dixon KW, Dixon KW, Kell SP, Barrett RL, Cribb PJ (2003). Propagation science, recovery and translocation of terrestrial orchids. Orchid conservation.

[CR23] Rasmussen HN (1995). Terrestrial orchids from seed to mycoheterotrophic plant.

[CR24] Rasmussen HN, Rasmussen FN (2009 Mar) Orchid mycorrhiza: implications of a mycophagous lifestyle. Oikos. 118:334–345

[CR25] Sarasan V, Cripps R, Ramsay MM, Atherton C, McMichen M, Prendergast G, Rowntree JK (2006). Conservation in vitro of threatened plants—progress in the past decade. In Vitro Cell Dev-Pl.

[CR26] Seaton PT, Pritchard HW (2003) Orchid germplasm collection, storage and exchange. ln: Dixon KW, Kell SP, Barrett RL, Cribb PJ (eds). Orchid conservation. Kota Kinabalu, Malaysia: Natural history publications (Borneo): 227-258

[CR27] Shefferson RP, Weiss M, Kull TIIU, Taylor DL (2005). High specificity generally characterizes mycorrhizal association in rare lady’s slipper orchids, genus *Cypripedium*. Mol Ecol.

[CR28] Swarts ND, Dixon KW (2009). Terrestrial orchid conservation in the age of extinction. Ann Bot.

[CR29] Taberlet P, Gielly L, Pautou G, Bouvet J (1991). Universal primers for amplification of three non-coding regions of chloroplast DNA. Plant Mol Biol.

[CR30] Taylor DL, McCormick MK (2008). Internal transcribed spacer primers and sequences for improved characterization of basidiomycetous orchid mycorrhizas. New Phytol.

[CR31] Tyson P (2000). The eighth continent: life, death and discovery in the lost world of Madagascar.

[CR32] UNEP-WCMC (Comps.) (2018) The checklist of CITES species website. CITES Secretariat, Geneva, Switzerland. Compiled by UNEP-WCMC, Cambridge, UK. http://checklist.cites.org. Accessed 18/10/2018

[CR33] Way MJ, Smith RD, Dickie JB, Linington SH, Pritchard HW, Probert RJ (2003). Collecting seed from non-domesticated plants for long-term conservation. Seed conservation: turning science into practice.

[CR34] White TJ, Bruns TD, Lee SB, Taylor JW, Innis MA, Gelfand DH, Sninsky JJ, White TJ (1990). Amplification and direct sequencing of fungal ribosomal RNA genes for phylogenetics. PCR protocols: a guide to methods and applications.

[CR35] Whitman M, Medler M, Randriamanindry JJ, Rabakonandrianina E (2011). Conservation of Madagascar’s granite outcrop orchids: the influence of fire and moisture. Lankesteriana.

[CR36] Wraith J, Pickering C (2018). Quantifying anthropogenic threats to orchids using the IUCN Red List. Ambio.

[CR37] Yokoya K, Zettler LW, Kendon JP, Bidartondo MI, Stice AL, Skarha S, Corey LL, Knight AC, Sarasan V (2015). Preliminary findings on identification of mycorrhizal fungi from diverse orchids in the Central Highlands of Madagascar. Mycorrhiza.

[CR38] Zelmer CD, Cuthbertson L, Currah RS (1996). Fungi associated with terrestrial orchid mycorrhizas, seeds and protocorms. Mycoscience.

[CR39] Zettler LW, Corey LL (2018). Orchid Mycorrhizal Fungi: isolation and identification techniques. Orchid propagation: from laboratories to greenhouses—methods and protocols.

[CR40] Zettler LW, Hofer CJ (1998). Propagation of the little club-spur orchid (*Platanthera clavellata*) by symbiotic seed germination and its ecological implications. Environ Exp Bot.

[CR41] Zettler LW, Stewart SL, Bowles ML, Jacobs KA (2001). Mycorrhizal fungi and cold-assisted symbiotic germination of the federally threatened eastern prairie fringed orchid, *Platanthera leucophaea* (Nuttall) Lindley. Am Midl Nat.

[CR42] Zettler LW, Poulter SB, McDonald KI, Stewart SL (2007). Conservation-driven propagation of an epiphytic orchid (*Epidendrum nocturnum*) with a mycorrhizal fungus. HortScience.

[CR43] Zettler LW, Rajaovelona L, Yokoya K, Kendon JP, Stice AL, Wood AE, Sarasan V (2017). Techniques for the collection, transportation, and isolation of orchid endophytes from afar: a case study from Madagascar. Bot Stud.

